# Supplementary material for: Research on the Interaction Mechanisms between ScCO2 and Low-Rank/High-Rank Coal with the ReaxFF-MD Force Field
Source: Molecules. 2024 Jun 25;29(13):3014. doi: 10.3390/molecules29133014 (PMC11243188; doi:10.3390/molecules29133014)
Supplement: Supplementary file 1 [file molecules-29-03014-s001.zip › molecules-3027236-supplementary.pdf]

Supplementary Materials

Research on the Interaction Mechanisms between ScCO2 and Low-Rank/High-Rank Coal with the ReaxFF-MD Force Field

Kui Dong[1] Shaoqi Kong[1,*]Zhiyu Niu[1] Bingyi Jia[2,3]

1 College of Mining Engineering, Taiyuan University of Technology, Taiyuan 030024, P. R. China
2 School of Safety Science and Engineering, Xi'an University of Science and Technology, Xi'an 710000, P. R. China
3 Xi’an Research Institute of China Coal Technology and Engineering Group Corp，Xi’an 710000, P. R. China
* Correspondence: kongshaoqi@tyut.edu.cn Tel: +86-13663611856

Table S1 Proximate and ultimate analyses of coal sample from YZ

$R_{o,max}$(%)	Promximate analysis (%)			Ultimate analysis (%)				
	M_{ad}	A_{ad}	V_{daf}	C	H	O	N	S
0.62	1.92	14.32	46.23	80.17	5.61	8.12	1.43	4.68

Table S2 Proximate and ultimate analyses of coal sample from CZ

$R_{o,max}$(%)	Promximate analysis (%)			Ultimate analysis (%)				
	M_{ad}	A_{ad}	V_{daf}	C	H	O	N	S
3.21	0.74	14.76	11.3	89.22	3.51	1.10	0.56	4.68

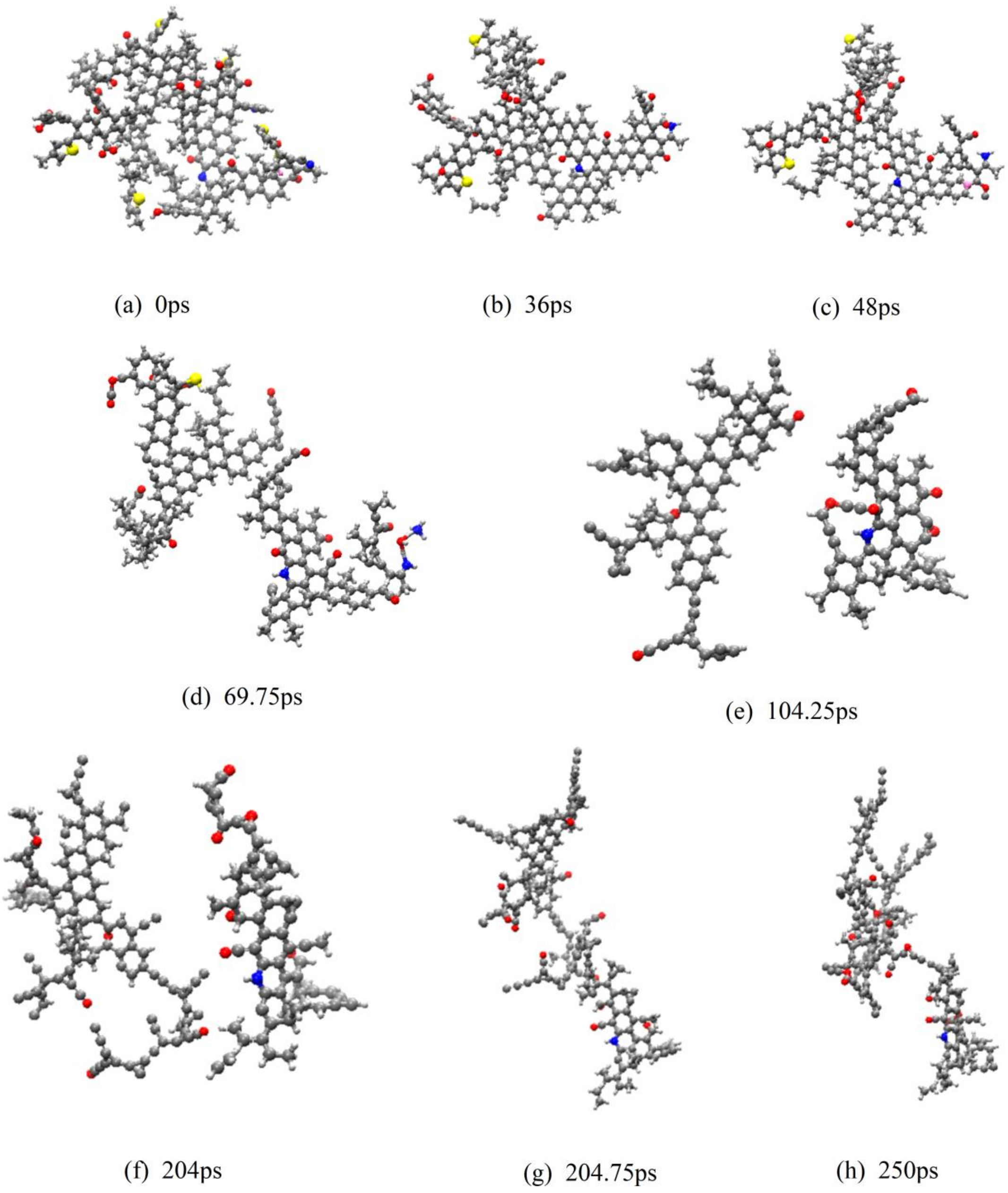

Figure S1. The low-rank coal macromolecular structures reaction process with $ScCO_2$

Pathway 1:Dehydrogenation reaction of -OH

① The hydrogen atom in the hydroxyl group is removed, forming a carbonyl radical (•C=O) and a water molecule (H_2O).

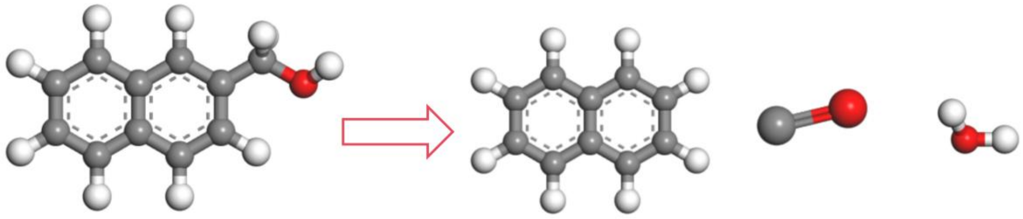

② The generated •C=O reacts with CO_2 to form CO and an intermediate of CO_3^{2-}

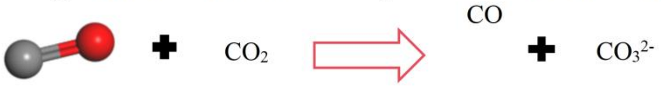

③ The carbonate ion (CO_3) may undergo reduction to form CO.

$$CO_3^{2-} \rightarrow CO + \cdot O$$

Pathway 2: Reduction reaction of -C=O-

The collision of H^+ results in the formation of H_2.The C=O in the carbonyl group is reduced by H_2 to a carbon-oxygen single bond (C-O), forming a hydroxyl group (-OH).

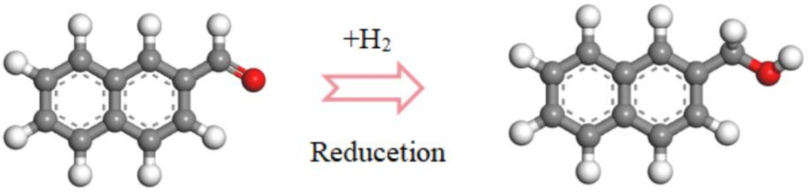

Pathway 3: Aromatization reaction of -C=O-

① The O atom in the -C=O- group is protonated, forming an intermediate of C^+ and ·OH.

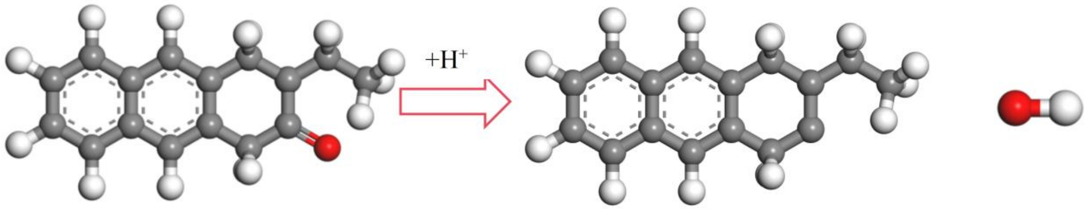

② C^+ reacts with a negatively charged methyl ion (CH_3^-) and surrounding carbon atoms through an addition reaction, forming surrounding carbon atoms.

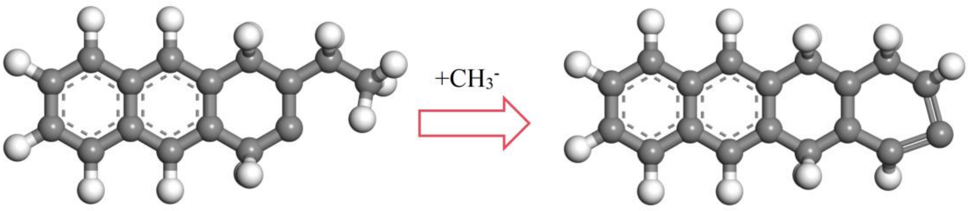

③ The cyclic structure undergoes rearrangement and dehydrogenation reactions to form the aromatic structure, simultaneously releasing H_2.

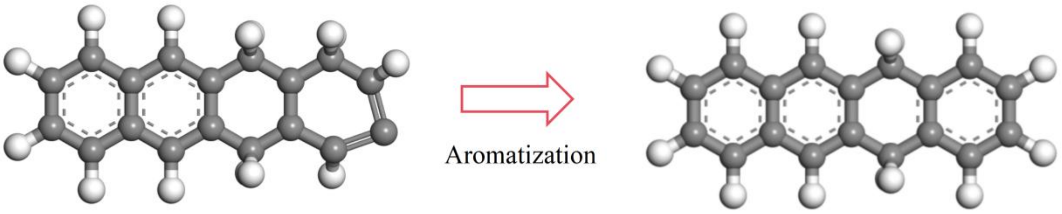

Pathway 4: Decomposition reaction of -COOH

The carboxyl group loses one oxygen atom to form a carbonyl group (C=O).

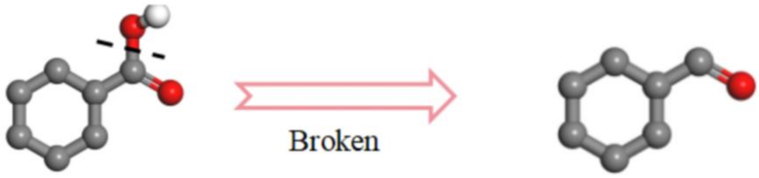

Pathway 5: Decomposition reaction of Aliphatic structure

• CH3 bonds in coal break, resulting in a series of hydrocarbons and gases.

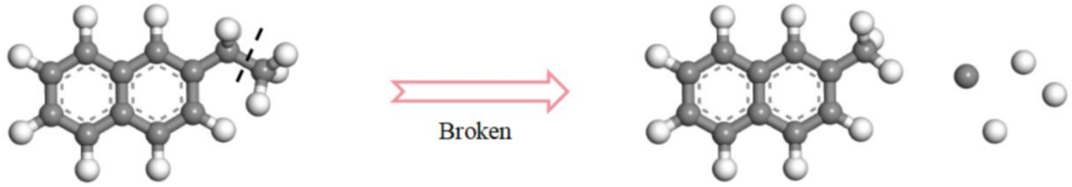

Pathway 6: Condensation reaction of Aliphatic structure

The methyl groups in coal undergo free radical reactions, leading to the generation of hydrocarbon compounds.

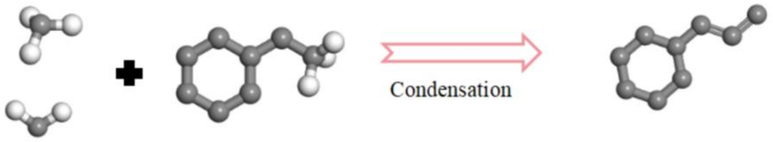

Pathway 7:Aromatic rings are broken directly

The π bonds of aromatic rings may be opened, forming radical intermediates,the radical intermediates can further react to form various small molecular compounds.

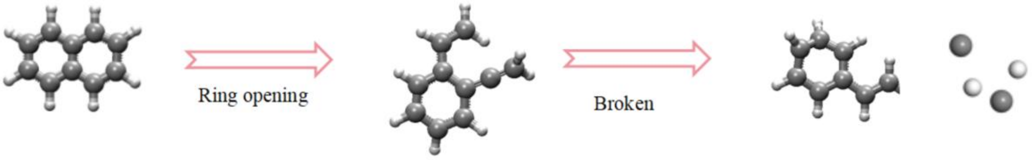

Pathway 8: Aromatic structures polymerize

The π bonds in the aromatic structure are broken, and the resulting radicals undergo polymerization reactions with CH_3^-, C^+, H^+, and other ions, forming long-chain hydrocarbons.

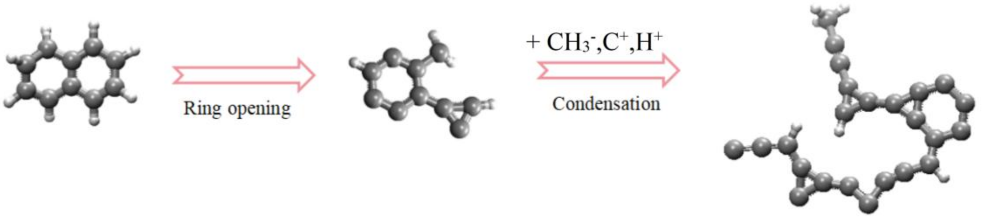

Pathway 9:Aromatic rings change from a six-membered aromatic ring to seven-membered ring, and then be damaged

① Ring expansion reaction:A carbon-carbon bond in the benzene ring can open, forming a reactive intermediate.A new carbon atom will be added to the benzene ring, forming a larger ring.

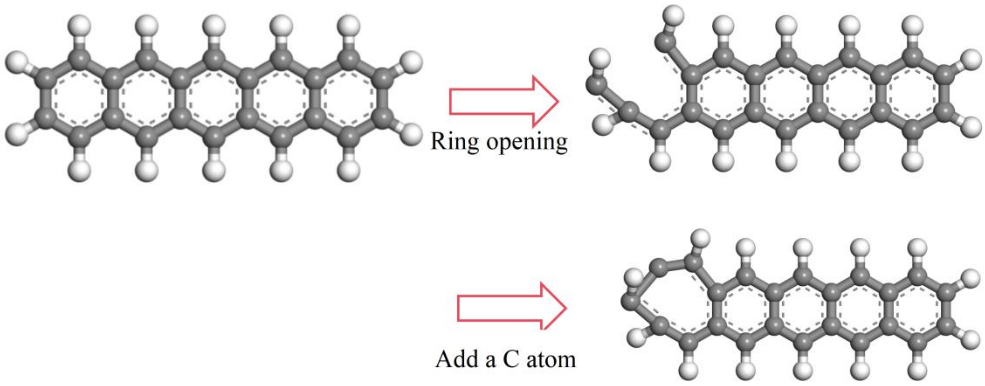

② Reorganization and rearrangement: After the formation of the new seven-membered ring, reorganization and rearrangement reactions occur to ensure the stability of the product.

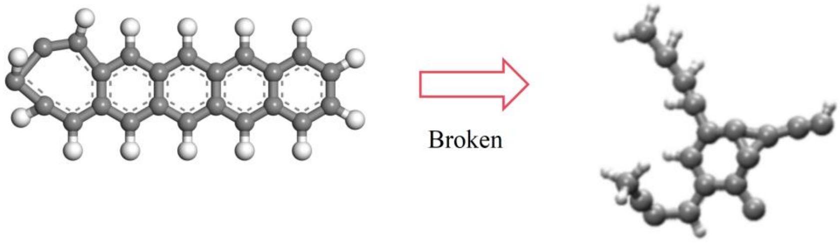

Pathway 10:Aromatic rings first break and then recombine

The π bonds in the aromatic structure are disrupted, leading to the cleavage of the aromatic ring. Subsequently, under the influence of radicals, the broken fragments recombine.

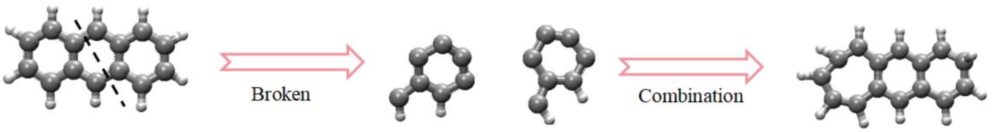

Figure S3 The reaction pathways detailed of $ScCO_2$ and coal

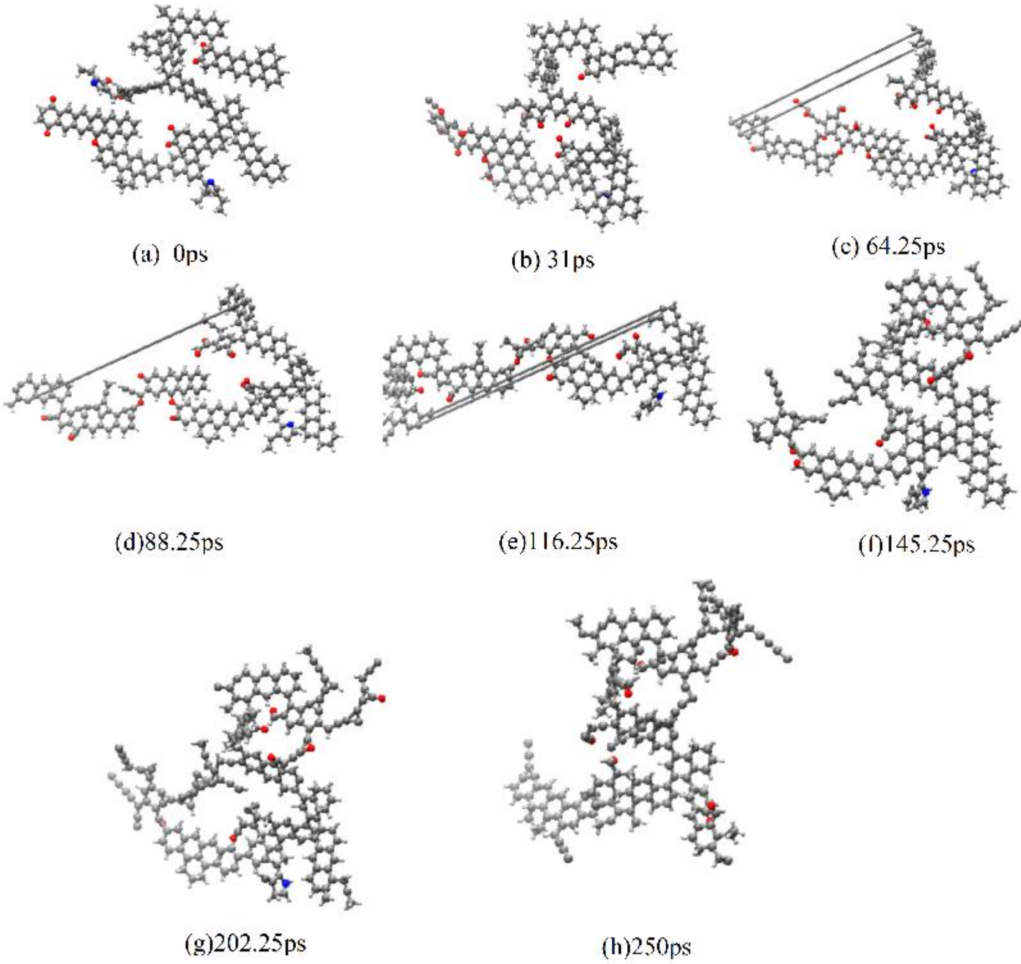

Figure S2. The high-rank coal macromolecular structures reaction process with $ScCO_2$
